# Targeting *LINC070974* inhibits lung adenocarcinoma cell proliferation and progression by interacting with Y-box binding protein 1: *LINC070974* and YBX1 regulate NSCLC

**DOI:** 10.3724/abbs.2024093

**Published:** 2024-06-20

**Authors:** Lin Liu, Pengfei Gong, Xueling Li, Li Zhang, Jiale Niu, Jinhui Zhu, Ziwei Wang, Xingwang Long, Tenghui Cao, Yewen Liu, Ganglin Wang, Tingming Fu, Liang Sun, Wei Li

**Affiliations:** 1 Key Laboratory of Laboratory Medicine Ministry of Education of China Zhejiang Provincial Key Laboratory of Medical Genetics School of Laboratory Medicine and Life Sciences Wenzhou Medical University Wenzhou 325035 China; 2 Zhuji Affiliated Hospital of Wenzhou Medical University Zhuji 311899 China; 3 College of Artificial Intelligence and Big Data for Medical Sciences Shandong First Medical University Jinan 250118 China; 4 School of Pharmacy Nanjing University of Chinese Medicine Nanjing 210023 China

**Keywords:** *LINC070974*, non-small cell lung cancer, Y-box binding protein 1, cyclinD1, nebulized inhalation

## Abstract

Non-small cell lung cancer (NSCLC) is the leading cause of cancer-related mortality worldwide. Increasing evidence suggests that long noncoding RNAs play crucial roles in lung cancer pathogenesis. We previously identified a novel lncRNA, *LINC070974*, which is associated with tumor cell proliferation. In the present study, we find that knockdown of *LINC070974* inhibits cell proliferation, migration and invasion as well as tumor formation both *in vitro* and in nude mice. *LINC070974* silencing also improves cisplatin efficacy in A549/DDP cells. The function of *LINC070974* may depend on its interaction with YBX1. Knockdown of *LINC070974* reduces the recruitment of YBX1 to the *CCND1* promoter and delays tumor progression through its coregulatory genes, which are mainly involved in the p53 signaling pathway. We utilize nebulized inhalation to deliver siRNAs targeting *LINC070974* and find that knockdown of *LINC070974* significantly prevents tumor metastasis and growth in lung tissues. These findings reveal the role of *LINC070974* in lung cancer and suggest a promising therapeutic approach involving siRNA inhalation.

## Introduction

Lung cancer is the leading cause of cancer-related mortality worldwide [1], accounting for approximately one-fourth of all cancer fatalities [ 2, 3], which can be classified into two main subtypes based on its histological morphology: small cell lung cancer (SCLC) and non-small cell lung cancer (NSCLC) [ 4, 5]. NSCLC is the predominant histological subtype, representing approximately 85% of all lung cancer cases [2]. Despite significant therapeutic advances in the treatment of NSCLC over the past two decades, the 5-year relative survival rate remains suboptimal at less than 15% [6].

Increasing evidence has demonstrated that long noncoding RNAs (lncRNAs) are a novel class of tumorigenic regulators that play crucial roles in the initiation, maintenance and progression of tumors through multiple mechanisms. Previous studies have revealed that lncRNAs can also serve as predictive, diagnostic and prognostic markers for lung cancer [ 7‒ 12]. For example, *LINC00472* was found to suppress cell migration and invasion in lung cancer [10]. *LINC00312* can induce the migration, invasion, and angiogenesis of lung cancer both *in vivo* and *in vitro* [12].

Herein, we identified the long intergenic noncoding RNA *LINC070974*, which is upregulated in lung adenocarcinoma tissues. We aimed to investigate the role of *LINC070974* in the growth and progression of lung cancer cells. We observed that knockdown of *LINC070974* significantly inhibited NSCLC cell proliferation, invasion and migration by inhibiting the recruitment of Y-box binding protein 1 (YBX1) to the *CCND1* promoter region. Notably, nebulized inhalation of an siRNA against *LINC070974* effectively suppressed metastasis and xenograft tumor growth. These findings suggest that *LINC070974* could become a promising and effective therapeutic target for treating NSCLC.

## Materials and Methods

### Oligos and vectors

All the siRNA oligos were synthesized by RiboBio (Guangzhou, China) and the oligo sequences are listed in Supplementary Table S1. Transfection was performed using Lipofectamine RNAiMAX (Thermo Fisher Scientific, Waltham, USA) according to the manufacturer’s protocols. The lentivirus plasmid pCDH was obtained from Weizhen Bioscience (Ji’nan, China), and the lentiviruses were prepared according to the manufacturer’s instructions, that is, the pCDH plasmid and a packaging vector (psPAX2 and pMD2. G) were co-transfected into HEK-293T cells. Then, the supernatant of the culture medium was collected and purified to isolate the lentivirus. The plasmid transformation was performed using Lipofectamin3000 (Thermo Fisher Scientific) according to the manufacturer’s protocol.

### Cell lines

The NSCLC cell lines A549, HCC827, and H1299 and human lung epithelial BEAS-2B cells were obtained from the Cell Bank of the Chinese Academy of Sciences (Shanghai, China). The cisplatin-resistant cell line A549/DDP (resistance index: 5.19) was purchased from Procell Biotechnology (Wuhan, China). The A549/DDP cell line was cultured in Ham’s F-12K medium (Procell) supplemented with 10% fetal bovine serum (Genimi, Calabasas, USA) and 1.5 μg/mL cisplatin. The other cell lines were cultured in Dulbecco’s modified Eagle’s medium (Gibco, Carlsbad, USA) supplemented with 10% FBS (Gemini) and 1% penicillin/streptomycin (Thermo Fisher Scientific) at 37°C and 5% CO _2_.

### Animal experiments

The animal experiments and animal care procedures used were approved by the Wenzhou Medical University Animal Care and Use Committee (No. wydw2021-0368). BALB/c nude mice (male, aged 4‒5 weeks) were purchased from Zhejiang Vital River (Hangzhou, China). Xenograft models were generated by subcutaneously injecting A549 cells (5 × 10 ^6^ cells) once and subjected to observation. After 8 weeks, the successfully implanted mice were divided into two groups for intratumoral siRNA injection (each with 2 nmol, once every 3 days, for a total of 10 injections). Another xenograft model was created by subcutaneously injecting sh-LINC07094- or sh-NC-transfected A549 cells, which were fed until 47 days after injection. For *in vivo* metastasis imaging, a mouse model was established by injecting A549 cells via the tail vein with luciferase plus sh-070974 or sh-NC. These mice were maintained for 45 days and subjected to imaging with an *in vivo* imaging system (IVIS; Lumina XR, Waltham, USA) by injecting fluorescein (Meilunbio, Dalian, China) at a dose of 200 mg/kg. For the nebulized inhalation test, mice were injected twice via the tail vein with A549 cells expressing luciferase, followed by inhalation of cholesterol-modified siRNA twice a week for 4 weeks for a total of 8 deliveries. Then, the mice were subjected to imaging. When the experiments were completed, the mice were euthanized, and the tumors and lungs were subjected to pathological analysis.

### Fluorescence *in situ* hybridization

The cells were fixed with 4% paraformaldehyde, permeabilized and hybridized with Cy3-labelled lncRNA probes (Riobio) according to the U6 method, and 18S RNA was used as the nuclear and cytoplasmic internal reference, respectively. The fluorescence signal was acquired by confocal microscopy and analyzed using software (Nikon, Tokyo, Japan).

### Colony formation, cell proliferation and viability assays

Colony formation assay was performed on a 6-well plate. Briefly, cells were seeded at a density of 2000 cells per well. After two weeks of culture, the cells were fixed with 4% paraformaldehyde, stained with 0.1% crystal violet and counted using ImageJ software (NIH, Bethesda, USA). Cell proliferation was detected using the CCK8 kit (Beyotime, Shanghai, China), and cell viability was tested using the MTT assay kit (Beyotime) according to the manufacturer’s protocol.

### Quantitative real-time PCR

Total RNA was extracted using Trizol (Invitrogen, Carlsbad, USA) and subjected to reverse transcription using the High-Capacity Reverse Transcription kit (Invitrogen). RT-qPCR reaction in One Step Plus Real-Time PCR system (Applied Biosystems, Foster City, USA) was performed on a Bio-Rad CFX96 instrument (Bio-Rad, Hercules, USA). The operation conditions were 95°C for 10 min, followed by 45 cycles at 95°C for 10 s and 60°C for 10 s. The relative RNA expression level was normalized to that of *β-actin* or *GAPDH* using the 2 ^–ΔΔCt^ method. All primer sequences are listed in Supplementary Table S1.

### Western blot analysis

Total protein was extracted using IP lysis buffer containing 1% PMSF (Beyotime), subsequently separated by SDS-PAGE, and transferred to PVDF membranes (Millipore, Billerica, USA). Then, the membrane was blocked with 5% BSA in Tris-buffered saline-Tween 20 (TBST) and incubated with primary antibodies (1:1000) at 4°C overnight, followed by incubation with HRP-labelled secondary antibodies (1:1000) for 90 min at room temperature. The results were visualized by enhanced chemiluminescence (ECL; Bio-Rad) and analyzed using ImageJ software. The antibody information is listed in Supplementary Table S2.

### Flow cytometry

A cell cycle assay was conducted using a cell cycle staining kit (MultiSciences, Hangzhou, China) according to the manufacturer’s protocol. Briefly, cells were collected and fixed with 70% ethanol at 4°C overnight. The cells were then treated with 500 μL of DNA staining solution for 30 min and analyzed by flow cytometry (Beckman Coulter Inc., Fullerton, USA).

### Wound healing and transwell assays

A wound healing assay was conducted on a 12-well plate with siRNA transfection for 24 h. The wound was formed using a 10-μL micropipette tip, and photos were taken at 0 h and 24 h. The wound area was calculated using ImageJ software. Transwell assays were carried out on a 24-well plate with transwell chambers (Corning, New York, USA) without Matrigel. The chamber was fixed with paraformaldehyde and stained with 0.1% crystal violet after the cells were incubated at 37°C for 24 h. The cells in the lower chamber were counted and photographed under a microscope (Nikon) in five random fields for analysis.

### RNA pull-down and mass spectrum

The RNA pull-down procedure was performed as previously described [13]. Briefly, lncRNA fragments were transcribed *in vitro* using the pGEM-3Z vector and T7 RNA polymerase RNA production system (Promega, Madison, USA). RNAs were purified using an RNeasy Mini kit (Qiagen, Valencia, USA), treated with DNase I (Quanta Biosciences, Beverly, USA), and subsequently labelled with biotin using T4 RNA ligase, followed by capture with streptavidin magnetic beads. The cells were lysed, extracted and mixed with magnetic beads. The beads were eluted to obtain the binding proteins. Then, the proteins were separated by SDS-PAGE and stained with silver staining reagent (Thermo Fisher Scientific). The differential bands were excised and detected by LC/MS (Orbitrap Fusion Lumos; Thermo Fisher Scientific) ( Supplementary Table S3), followed by alignment with a database to identify the proteins.

### RNA immunoprecipitation

RNA immunoprecipitation (RIP) was conducted using a RIP kit (Millipore) according to the manufacturer’s protocol. Briefly, cells were lysed, and total protein was extracted, followed by immunoprecipitation with an anti-YBX1 antibody (diluted 1:50; Millipore) combined with protein A/G magnetic beads. Next, the complexes were immobilized with a magnet stand, washed to remove unbound substances, subjected to RNA extraction, and finally quantified by RT-qPCR.

### RNA sequencing and transcriptomic analysis

Total RNA was extracted using Trizol reagent (Invitrogen) and subjected to sequencing by BGI Genomics (Shenzhen, China). This step was followed by quality control, mRNA enrichment, cDNA synthesis, and the construction of sequencing libraries. Subsequently, each sample underwent sequencing with a depth of 10 million reads. The acquired reads were aligned and assembled against transcripts, filtered and normalized among samples. The transcripts among groups were then subjected to statistical analysis for differential gene expression.

The sequencing data were stored in and processed through the BGI cloud server ( https://biosys.bgi.com). Genes were considered significantly differentially expressed if the *P* value was < 0.05 and the log _2_FC was > 1. The functional enrichment analysis included Kyoto Encyclopedia of Genes and Genomes (KEGG) and Gene Ontology (GO) enrichment analyses, which are displayed as bubble charts. Some of the DEGs involved in the p53 signaling pathway are presented as heatmaps ( Supplementary Table S4).

### Chromatin immunoprecipitation

The ChIP assay was conducted using a chromatin immunoprecipitation kit (CST, Beverly, USA). According to the manufacturer’s manual, the procedure includes crosslinking proteins to DNA and cell lysis and chromatin fragmentation by sonication. Subsequently, immunoprecipitation was performed using an anti-YBX1 antibody (dilution 1:50; Millipore), and the precipitates were washed for subsequent analyses. Then, the DNA was purified and analyzed via RT-qPCR.

### Hematoxylin-eosin staining

The lung tissues were harvested and fixed in 4% formaldehyde overnight, dehydrated in 70% ethanol, cleared in xylene and embedded in paraffin. Lung tissues were cut into 5-μm-thick sections, dewaxed with xylene and stained with hematoxylin-eosin (HE). Pathological sections were observed under a light microscope (Nikon), and the results were evaluated by a pathologist.

### Bioinformatics

*LINC070974* and YBX1 expression data and clinical information were downloaded from the TCGA database ( https://portal.gdc.cancer.gov/). The expression data of 59 normal samples and 535 LUAD samples were obtained to analyze *LINC070974* expression, of which 54 samples included both tumor tissues and paracancerous tissues. A total of 58 normal tissues and 405 LUAD tissues were used to investigate YBX1 expression. The survival data of 247 patients with high YBX1 expression and 259 patients with low YBX1 expression were utilized for 5-year survival rate analysis. The correlation between *LINC070974* and YBX1 expression was analyzed in the TCGA database.

### Statistical analysis

Statistical analysis was performed using GraphPad Prism 8.3.0 (GraphPad Software, San Diego, USA). Data are presented as the mean  ±  standard errors. Student’s *t* test was performed to compare two groups, and one-way ANOVA was used to determine the significant differences among three or more independent groups. Differences were considered to be statistically significant when the *P* value was less than 0.05.

## Results

### Knockdown of *LINC070974* inhibits NSCLC cell proliferation *in vitro*

**Figure 1:**
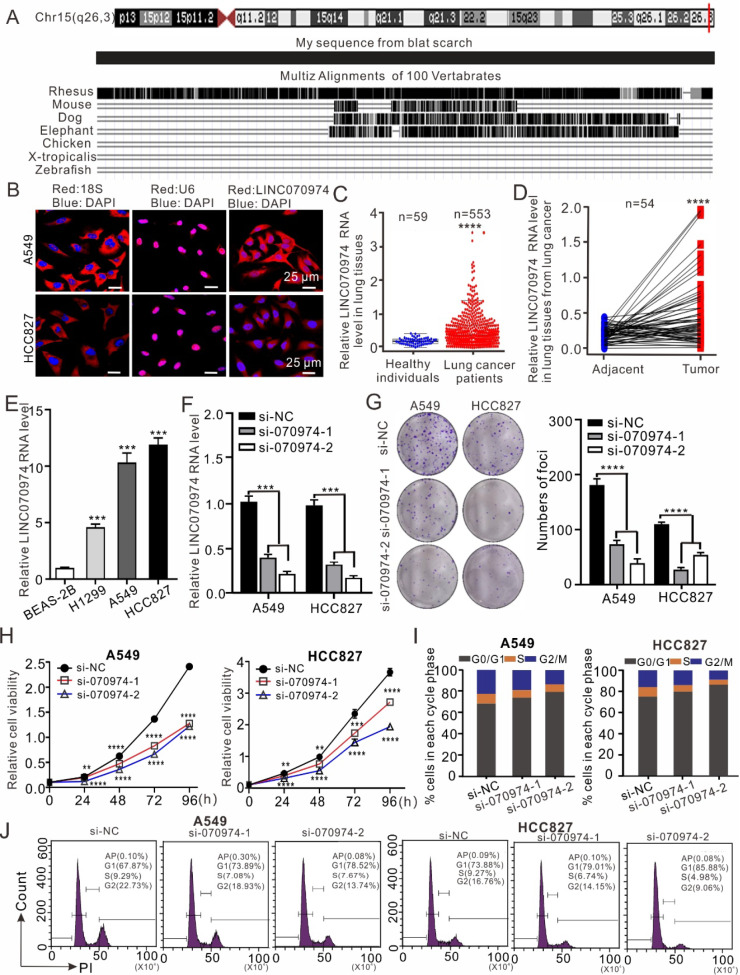
Figure 1 *LINC070974* characteristics and its ability to inhibit NSCLC cell proliferation and cell cycle (A) Chromosome localization and conservation analysis. (B) Subcellular localization by FISH. (C) Differential expression of LINC070974 between healthy controls and individuals with lung cancer according to TCGA data. (D) Paired comparison of LINC070974 expression between tumor and adjacent tissues from TCGA data. (E) LINC070974 expression in the cell lines BEAS-2B, H1299, A549 and HCC827 was determined by RT-qPCR. (F) Validation of the designed siRNA oligos. (G) Colony formation assay. (H) Cell viability determined by CCK-8 assay. (I,J) Cell cycle distribution analysis by flow cytometry in A549 and HCC827 cells with LINC070974 knockdown. si-070974 represents LINC070974 knockdown; si-NC represents scrambled siRNA. **P < 0.01, ***P < 0.001, ****P < 0.0001.

We previously identified one novel long noncoding RNA, *LINC070974*, which was authorized for this study (ZL202011504636.1). *LINC070974* is located in the human chromosome 15q26.3 region and is conserved among primates but exhibits limited homology with species beyond this taxonomic group ( Figure 1A). LNC070974 is distributed both in the nucleus and cytoplasm but is predominantly located in the cytoplasm, particularly in HCC827 cells ( Figure 1B).

We investigated the expression of *LINC070974* by utilizing the TCGA-LAUD database and observed that the level of *LINC070974* was significantly greater in the lung tissues of patients than in those of healthy individuals ( Figure 1C) and in tumors than in adjacent tissues ( Figure 1D). The expression of *LINC070974* was also significantly elevated in the human NSCLC cell lines H1299, A549 and HCC827 when the control cell line BEAS-2B was used as a reference, especially in the A549 and HCC827 cell lines ( Figure 1E).

We designed two siRNAs to silence *LINC070974* in A549 and HCC827 cells ( Figure 1F). SiRNA-mediated knockdown of *LINC070974* significantly reduced colony formation in both A549 and HCC827 cells ( Figure 1G) and decreased cell viability ( Figure 1H). Moreover, silencing of *LINC070974* increased the ratio of cells in the G1 phase and decreased the ratio of cells in the S and G2/M phases ( Figure 1I,J).

Cisplatin resistance is the primary cause of chemotherapy failure in practice [14]. We further used the cisplatin-resistant cell line A549/DDP ( Supplementary Figure S1A,B) to explore the role of *LINC070974*. *LINC070974* was also highly expressed in A549/DDP cells ( Supplementary Figure S1C), and knockdown of *LINC070974* decreased A549/DDP cell colony formation and reduced cell viability ( Supplementary Figure S1D‒F), even after cisplatin treatment ( Supplementary Figure S1G,H). These results suggest that silencing of *LINC070974* improves cisplatin resistance in lung cancer.

### Knockdown of *LINC070974* inhibits tumor growth in xenograft mice

**Figure 2:**
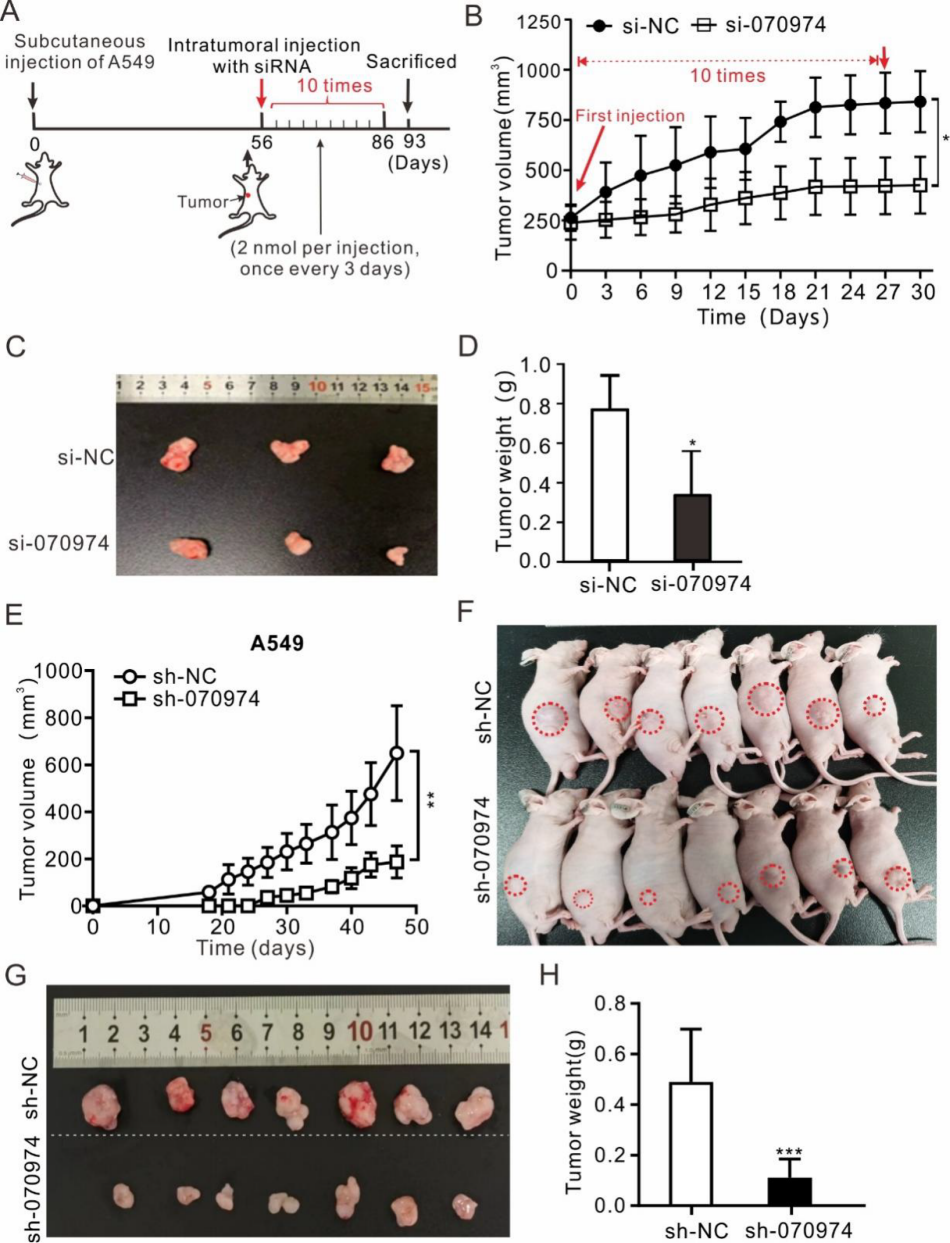
Figure 2 *LINC070974* knockdown delays tumor growth in xenograft nude mice (A) Timeline of A549 subcutaneous injection and siRNA intratracheal treatment in nude mice. (B) Tumor volume changes during siRNA treatment in xenograft nude mice. (C) Excised tumor size measurement. (D) Comparison of tumor weight. (E) Tumor volume changes in xenograft nude mice injected with sh-070974-treated A549 cells. (F) Mice bearing tumors at the end of the experiment. (G) Images showing the excised tumor size. (H) Comparison of tumor weight between the sh-070974 and sh-NC groups. sh-070974 represents a cell line in which LINC070974 was stably expressed via lentivirus-mediated shRNA, and sh-NC represents scrambled shRNA. *P < 0.05, **P < 0.01, ***P < 0.001.

To further validate that *LINC070974* suppresses tumor growth, we subcutaneously transplanted tumor cells into nude mice. As shown in Figure 2A, A549 cells were injected subcutaneously, followed by successive siRNA injections into the tumors. After 10 times of siRNA deliveries, the volume and weight of the tumors were significantly lower in the si-070974-treated group than in the control group ( Figure 2B‒D).

Moreover, we constructed a stable *LINC070974*-shRNA-mediated *LINC070974*-knockdown cell line via lentiviral transduction and then subcutaneously injected into BALB/C nude mice. We found that tumor volume increased more slowly in the si-070974 group than in the si-NC control group over time ( Figure 2E,F). On the 47 ^th^ day, the mice were sacrificed, and the tumors were isolated for analysis. We found that the tumors in the si-070974 group significantly decreased in size ( Figure 2G) and that the weights of the xenograft tumors in the si-070974 group were less than those in the control sh-NC group ( Figure 2H).

### Silencing of *LINC070974* inhibits cell migration and invasion *in vitro* and metastasis *in vivo*

**Figure 3:**
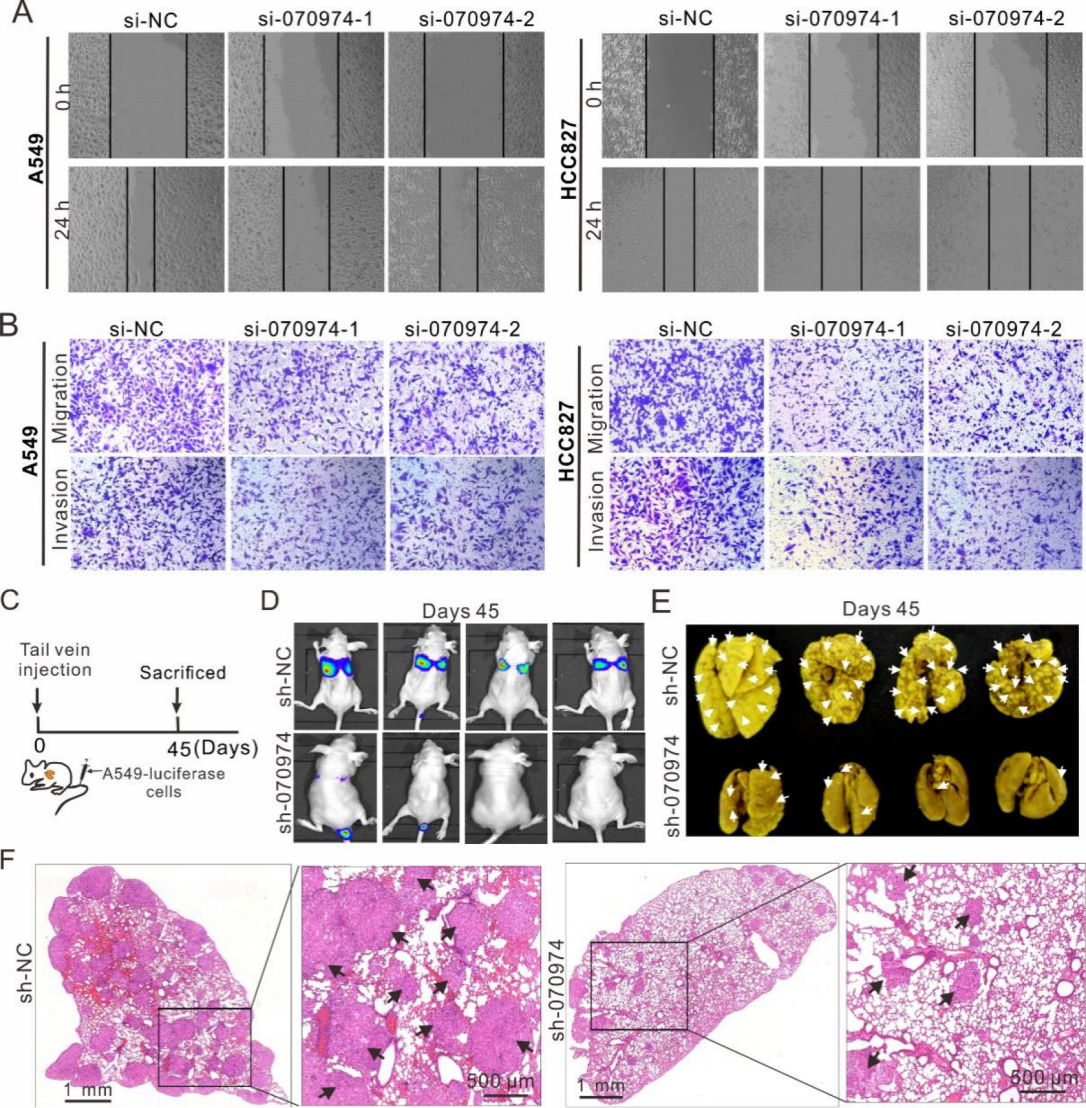
Figure 3 *LINC070974* knockdown inhibits cell migration and invasion *in vitro* and suppresses metastasis *in vivo* (A) Wound healing and (B) Transwell assays in A549 and HCC827 cells with LINC070974 knockdown. (C) Timeline illustration of the tail vein injection of luciferase-expressing A549 cells. (D) Mice were subjected to live luciferase imaging on the 45th day after luciferase-expressing A549 cells were injected. (E) Excised lungs with fixation and tumor nodules. (F) Hematoxylin-eosin staining of lung tissues and tumor nodule observation. Arrows indicate the tumor nodules.

To determine the role of *LINC070974* in tumor progression, we performed wound healing and Transwell assays to assess cell migration and invasion, respectively. The results showed that knockdown of *LINC070974* significantly delayed the migration and invasion of both the A549 and HCC827 cell lines ( Figure 3A,B and Supplementary Figure S2A,B).

We further constructed a luciferase-expressing A549 cell line and injected these cells into mice via the tail vein ( Figure 3C ). After 45 days, we examined the mice by bioluminescence *in vivo* imaging and found a substantial decrease in luciferase signal strength in the lungs of the sh-070974 group compared to the sh-NC group ( Figure 3D). Morphological and histochemical analyses revealed fewer tumor nodules in the lungs of the sh-070974 group than in those of the sh-NC group ( Figure 3E,F and Supplementary Figure S2C).

### *LINC070974* interacts with YBX1 to regulate cell proliferation, migration and invasion

**Figure 4:**
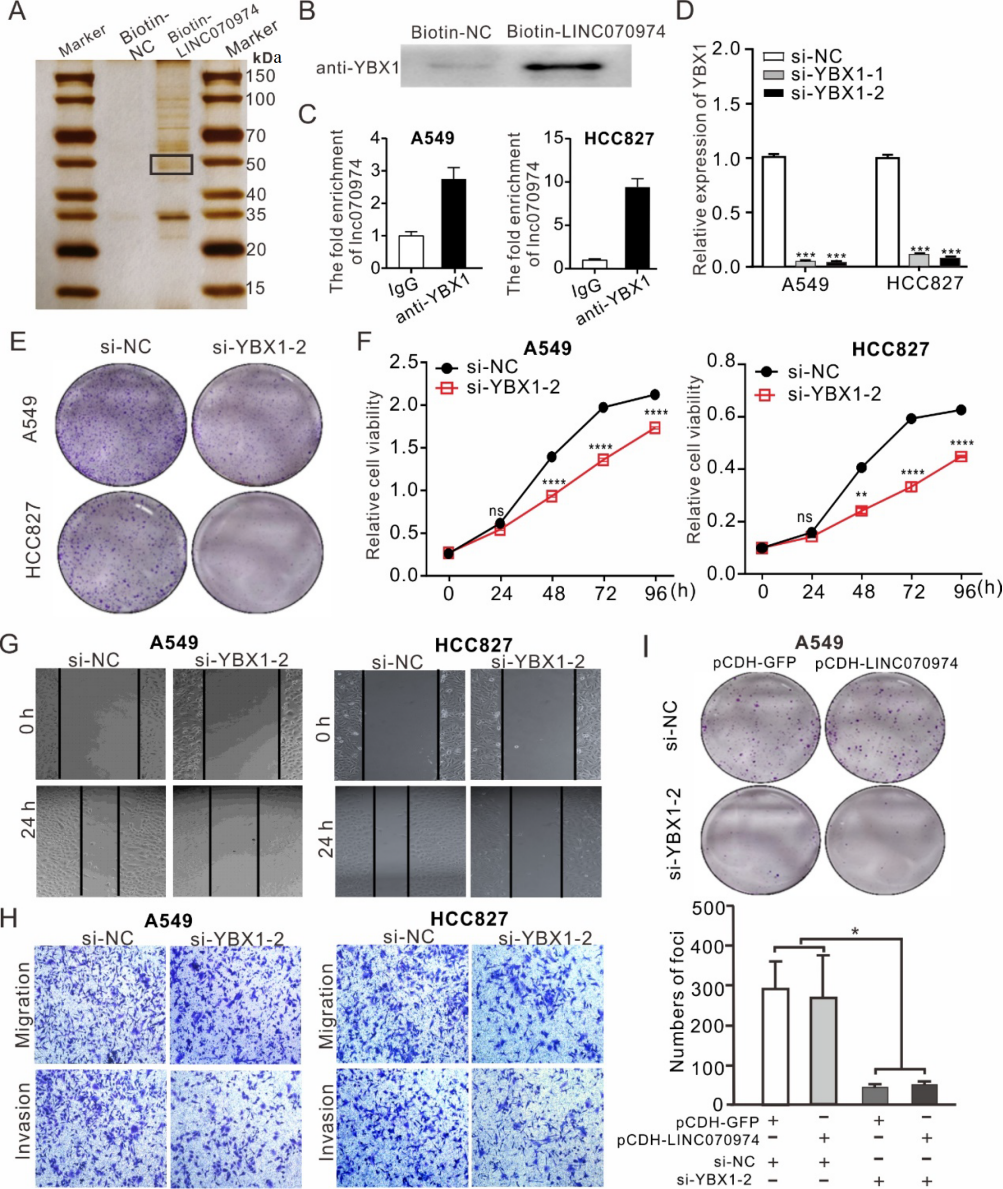
Figure 4 YBX1 is a *LINC070974*-binding protein that regulates NSCLC cell proliferation, migration and invasion (A) Differential bands identified by SDS-PAGE with silver staining following biotin-LINC070974 RNA pull-down. (B) Validation of the RNA pulldown protein YBX1 by western blot analysis. (C) Reversal of the LINC070974-YBX1 interaction was verified by a RIP assay using an anti-YBX1 antibody. (D) Design and validation of siRNAs targeting YBX1 RNA. (E) Colony formation assay. (F) Cell viability determined by the CCK-8 assay. (G) Wound healing assay. (H) Transwell assay of YBX1-knockdown A549 and HCC827 cell lines. (I) Colony formation ability of pCDH-LINC070974-treated A549 cells with YBX1 knockdown. *P < 0.05, **P < 0.01, ***P < 0.001, ****P < 0.0001. ns means not statistically significant.

To elucidate the intracellular mechanism of action of *LINC070974*, we performed an RNA pull-down assay to identify *LINC070974*-interacting proteins. As shown in Figure 4A, an ~50 kDa distinct band appeared in the biotin- *LINC070974* precipitate. The band was cut and then subjected to analysis by mass spectrometry ( Supplementary Table S3). Among these identified proteins, YBX1, an RNA-binding protein associated with tumor cell proliferation, progression and metastasis [ 15‒ 17], was validated by western blot analysis using an anti-YBX1 antibody ( Figure 4B) and RNA binding protein immunoprecipitation assay ( Figure 4C). These results confirmed the interaction between *LINC070974* and YBX1.

Utilizing the TCGA database, we found that YBX1 expression is significantly upregulated in lung cancer patients compared with healthy individuals and is positively correlated with overall survival (OS) in lung cancer patients ( Supplementary Figure S3A,B). Thus, we knocked down *YBX1* in A549 and HCC827 cells ( Figure 4D) to observe colony formation and cell viability, and found that both were reduced ( Figure 4E,F and Supplementary Figure S3C). In addition, the wound healing and transwell assays demonstrated that *YBX1* knockdown also suppressed cell migration and invasion ( Figure 4G,H and Supplementary Figure S3D,E), consistent with the results of *LINC070974* silencing.

Moreover, we investigated the interdependence between *LINC070974* and YBX1. The TCGA-LUAD dataset revealed that there was a positive correlation between *LINC070974* and YBX1 RNA expression ( Supplementary Figure S3F). *LINC070974* knockdown had no significant effect on the expression of YBX1 at either the RNA or protein level ( Supplementary Figure S3G,H). However, the colony formation assay showed that cell proliferation was inhibited by si-YBX1 in pCDH-LINC070974-overexpressing A549 cells ( Figure 4I and Supplementary Figure S3I), which suggested that *LINC070974* depends on YBX1 to regulate cell proliferation, migration and invasion *in vitro*.

### Transcriptomic analysis reveals the coregulatory network of *LINC070974* and YBX1

**Figure 5:**
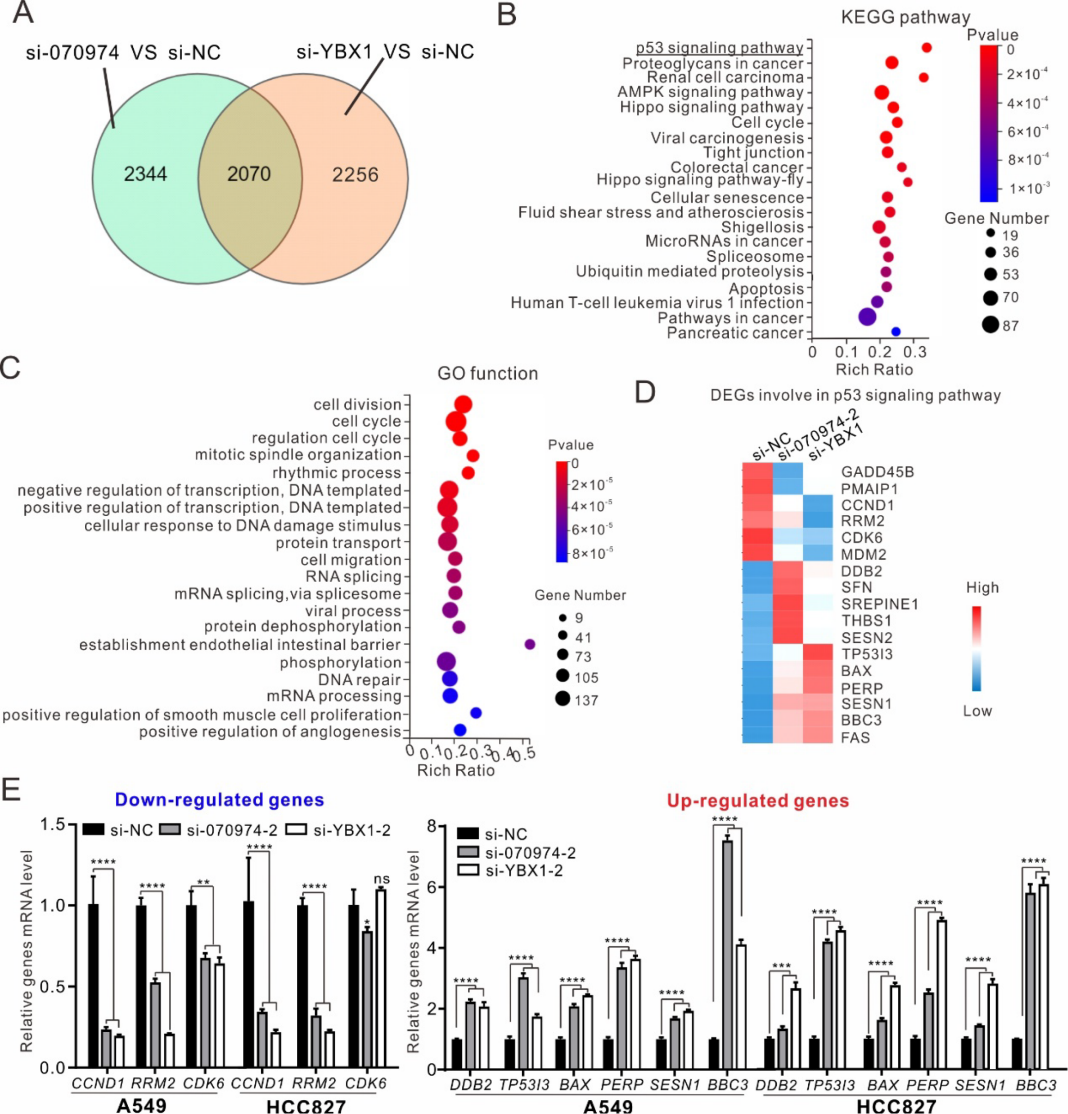
Figure 5 RNA sequencing and transcriptomic analysis of NSCLC cells with silencing of *LINC070974* and *YBX1* (A) Differentially expressed gene sets among the si-LINC070974, si-YBX1 and si-NC groups. (B,C) KEGG pathway enrichment and GO functional analysis of DEGs from the intersection. (D) Heatmap clustered by representative DEGs involved in the p53 signaling pathway. (E) Validation of p53 pathway-related gene expression by RT-qPCR. *P < 0.05, **P < 0.01, ***P < 0.001, ****P < 0.0001. ns represents no statistical significance.

To interpret the coregulatory network in NSCLC, we performed transcriptome analysis of both *LINC070974*- and YBX1-knockdown A549 cells through RNA sequencing. A total of 4,414 DEGs between the si-070974 and si-NC groups and 4,326 genes between the si-YBX1 and si-NC groups were identified in A549 cells by alignment with the reference genome ( Supplementary Table S4-1,2). We further screened 2070 genes associated with the intersection between the “si-070974 vs si-NC” and “si-YBX1 vs si-NC” sets ( Figure 5A and Supplementary Table S4-3). Furthermore, we selected 1536 of 2070 genes with identical up- or downregulation tendencies for KEGG and GO analysis ( Supplementary Table S4-4). These genes were classified into the p53 signaling pathway, proteoglycans in cancer, renal cell carcinoma, *etc*., through KEGG pathway analysis ( Figure 5B) and were functionally enriched in cell division, the cell cycle, regulation of the cell cycle, *etc*., according to Gene Ontology (GO) functional analysis ( Figure 5C).

To determine the crucial genes involved in the coregulation of these genes, we adopted more stringent analytical conditions, setting the thresholds to *P*  < 0.01 and |log _2_FC| ≥ 2. We identified 17 genes enriched in the p53 signaling pathway ( Supplementary Table S4-5) to construct a heatmap ( Figure 5D) and used qPCR to confirm the expression levels of these genes, including the downregulated genes *CCND1*, *RRM2* and *CDK6* and the upregulated genes *DDB2*, *TP53I3*, *BAX*, *PERP*, *SESN1* and *BBC3*, in si-070974- and si-YBX1-treated NSCLC cells ( Figure 5E).

### *LINC070974* cooperates with YBX1 to regulate cyclin D1 and promotes YBX1 recruitment to the *CCND1* promoter

**Figure 6:**
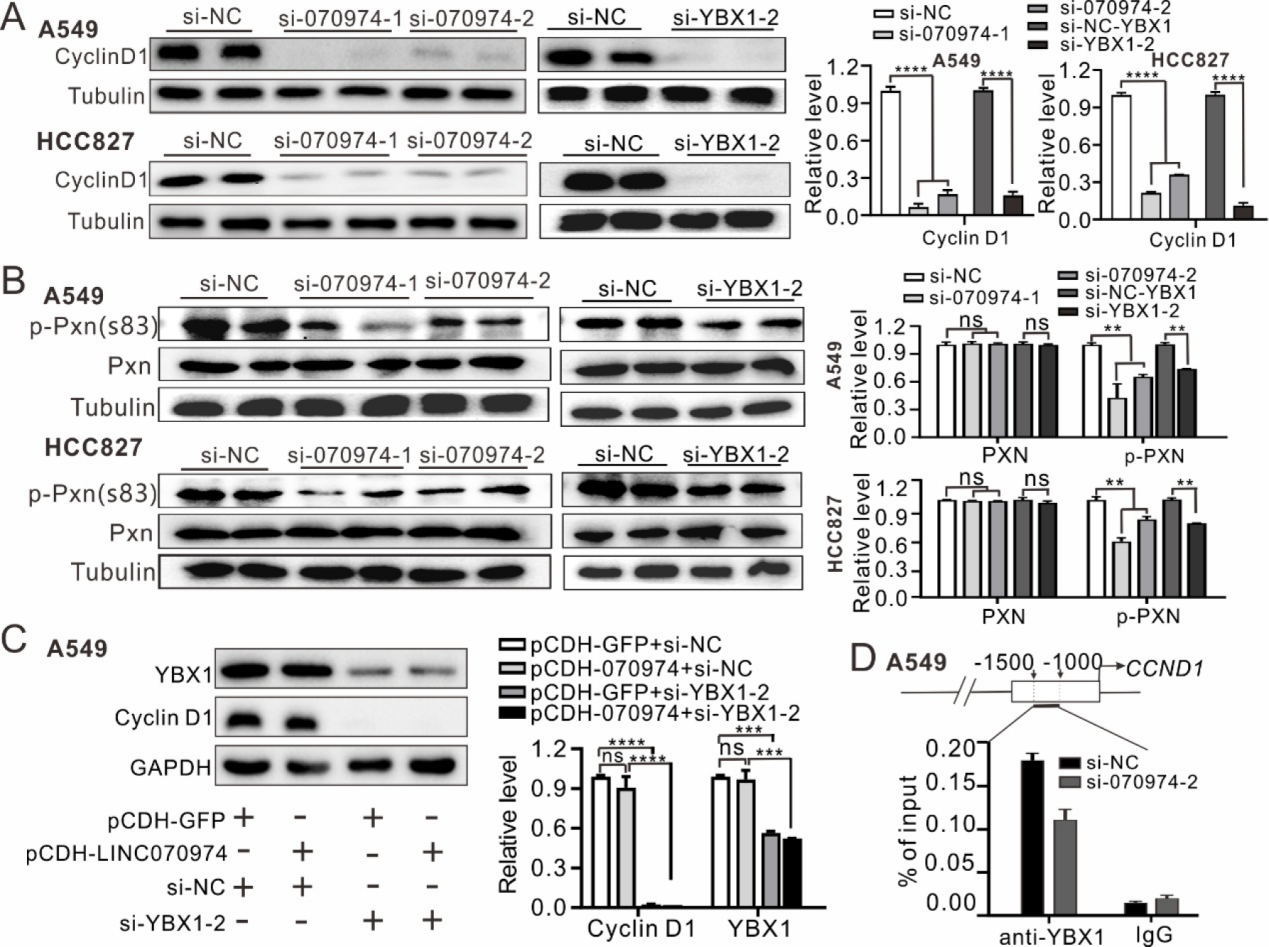
Figure 6 Cyclin D1 is regulated by either *LINC070974* or YBX1 via *LINC070974*, which promotes YBX1 binding to the *CCND1* promoter (A,B) Cyclin D1 content and paxillin and p-paxillin (Ser83) levels in A549 and HCC827 cells with LINC070974 or YBX1 knockdown determined by western blot analysis. (C) YBX1 and cyclin D1 levels in LINC070974-overexpressing A549 cells with/without si-YBX1. (D) Quantitative analysis of YBX1 binding to the CCND1 promoter by ChIP-PCR. **P < 0.01, ***P < 0.001, ****P < 0.0001. ns represents not statistically significant.

Of the above differentially expressed genes, *CCND1* encodes the protein cyclin D1, which is crucial for cell cycle regulation. The protein levels of cyclin D1 decreased significantly with the knockdown of *LINC070974* and *YBX1* in the A549 and HCC827 cell lines ( Figure 6A). The expression levels of cyclin D1 downstream genes, including *CCNE1*, *PCNA*, *POLA1* and *TK1*, were also significantly decreased in A549 and HCC827 cells with *LINC070974* knockdown ( Supplementary Figure S4A). Cytoplasmic cyclin D1 has been reported to regulate cell invasion and metastasis by phosphorylating paxillin (PXN) [18]. In this study, we found that the phosphorylation of PXN at Ser83 was significantly decreased in both *LINC070974-* and *YBX1*-knockdown cells ( Figure 6B). Notably, overexpression of *LINC070974* had no effect on the protein level of YBX1 or cyclin D1, nor rescued the reduction in cyclin D1 expression resulted from *YBX1* knockdown ( Figure 6C).

To elucidate the mechanism by which *LINC070974* facilitates YBX1-mediated gene expression regulation, we identified the binding position of YBX1 to the promoter of *CCND1*, which ranged from –1000 nt to –1500 nt, by ChIP-PCR assay ( Supplementary Figure S4B). Moreover, we found that knockdown of *LINC070974* significantly reduced the binding of YBX1 to this region, indicating that *LINC070974* might promote the recruitment of YBX1 to the *CCND1* promoter ( Figure 6D).

### Inhalation of *LINC070974* siRNA oligos effectively inhibits the metastasis of lung cancer cells

**Figure 7:**
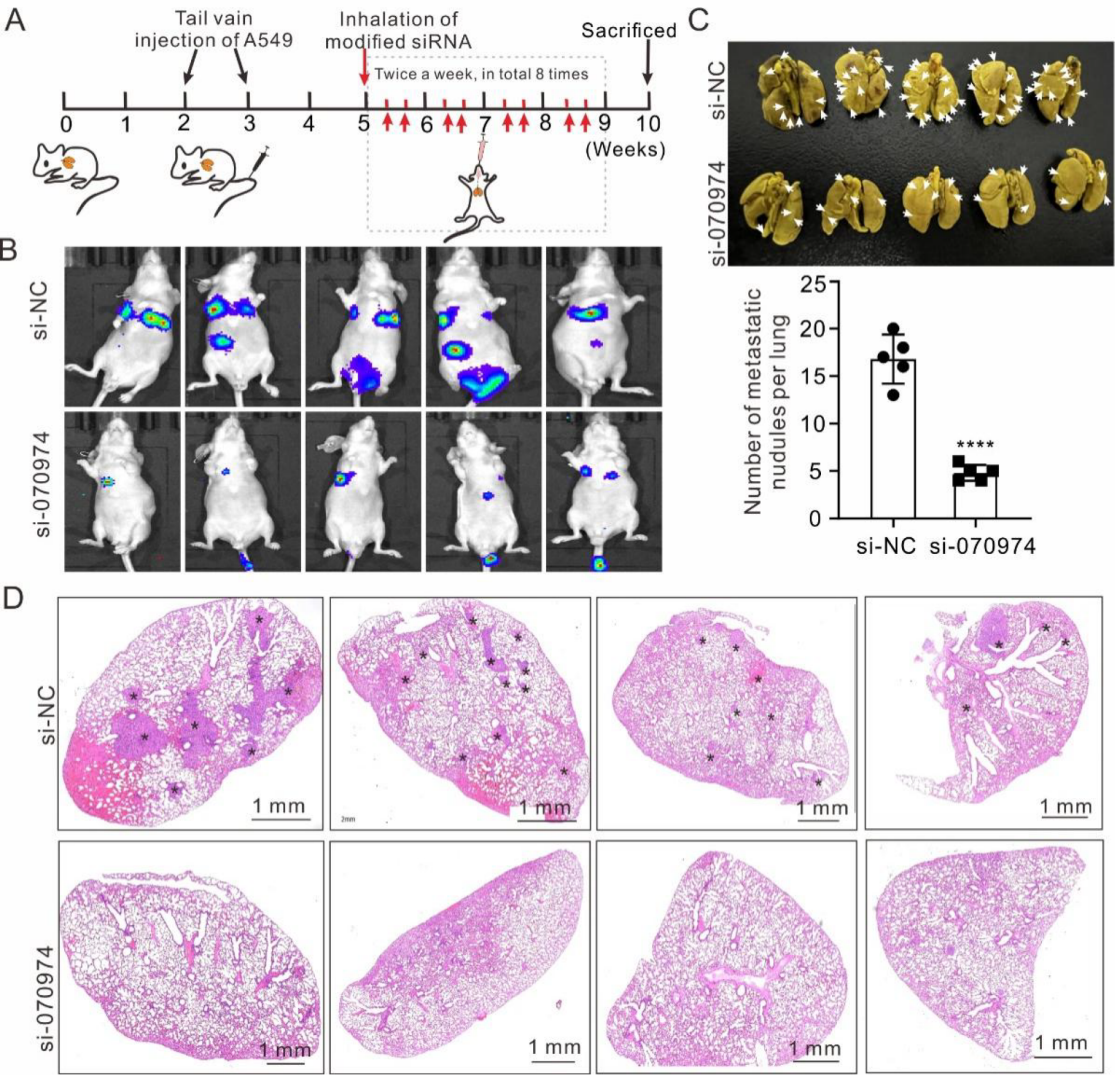
Figure 7 Nebublized inhalation of siRNAs targeting *LINC070974* prevents NSCLC cell metastasis and growth in the lungs (A) Timeline illustration of mouse preparation by tail vein injection and treatment with siRNA inhalation. (B) Live luciferase imaging of mice with nebulized inhalation of siRNA. (C) Excised lungs with fixation and tumor nodule counting. (D) H&E staining of lung tissues to observe tumor nodules. Arrows indicate the tumor nodules. ****P < 0.0001.

Inhalation administration is a promising noninvasive treatment for lung diseases. To explore the potential of *LINC070974* as a therapeutic target for treating NSCLC, we delivered a siRNA (modified by cholesterol conjugation) targeting *LINC070974* to NSCLC mice via nebulized inhalation ( Figure 7A). Compared with that in the si-NC group, the luciferase signal in the si- *LINC070974* group was significantly decreased ( Figure 7B). Morphological ( Figure 7C) and histochemical ( Figure 7D) analyses of lung tissues also revealed a significant decrease in the number of tumor nodules in mice treated with *LINC070974* siRNA compared with those in the si-NC group, suggesting that siRNA inhalation could effectively inhibit the expression of target lncRNAs in the lungs.

## Discussion

We identified a novel lncRNA, *LINC070974*, which has been authorized for this study. It is significantly upregulated in lung cancer tissues according to TCGA data. We performed *LINC070974* or *YBX1* knockdown experiments in A549 and HCC827 cells and found that *LINC070974* downregulation significantly inhibited cell proliferation, migration and invasion. *LINC070974* knockdown also inhibited the growth of cisplatin-resistant A549/DDP cells. We further confirmed the effect of *LINC070974* knockdown using a xenograft tumor mouse model.

This study demonstrated that *LINC070974* inhibits tumor cell growth and migration in a YBX1-dependent manner. YBX1 has been reported to regulate cellular functions such as transcription, posttranscriptional splicing and RNA stability maintenance [ 19, 20]. Additionally, YBX1 is involved in promoting tumor progression and drug resistance [21]. Recent studies have reported that a variety of lncRNAs exhibit binding affinity for YBX1 and exert different effects on tumor cells. For example, the regulatory effect of *HOXC*- *AS3* on HDAC5 expression is mediated through its interaction with YBX1, which facilitates the proliferation and migration of gastric cancer cells [22]. The *DARS1*- *AS1*/YBX1 complex elevates the expression of the key tumorigenesis genes *E2F1* and *CCND1* in glioblastoma stem cell-like cells [23]. The lncRNA *MIR22HG* interacts with YBX1 and inhibits p21 expression [24]. Therefore, targeting specific lncRNAs could selectively affect the function of YBX1 in certain tumor tissues, suggesting an approach for cancer target therapy [25].

**Figure 8:**
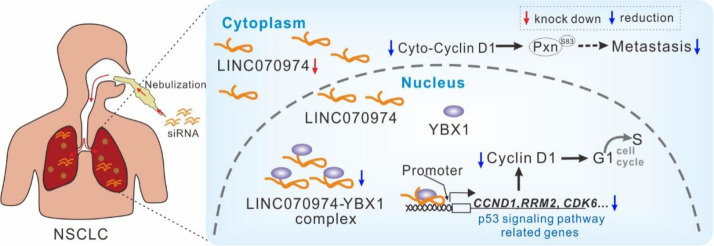
Figure 8 *LINC070974* silencing inhibits the progression of NSCLC via its interaction with YBX1 LINC070974 is highly expressed in NSCLC cells and binds to YBX1. Silencing of LINC070974 delays YBX1 binding to promoters, and this reduces the expressions of genes involved in the p53 pathway, such as CCND1 (encoding cyclinD1). Finally, LINC070974 silencing inhibits tumor proliferation, migration, invasion and metastasis.

We found that *LINC070974* silencing retards YBX1 binding to the promoter region of the *CCND1* gene, leading to a significant reduction in cyclin D1 level. To further elucidate the coregulatory effects of *LINC070974* and YBX1 on gene expression, we examined the intersection of DEGs between the si- *LINC070974*-transfected cells and si-NC-transfected cells and between the si-YBX1-transfected cells and si-NC-transfected cells. The intersection genes were significantly enriched in the p53 signaling pathway, which functions in cell cycle regulation. Notably, we observed significant downregulation of the *CCND1*, *RRM2* and *CDK6* genes, which are known oncogenes [26], in the si- *LINC070974* and si-YBX1 cell lines, whereas the *DDB2* and *TP53I3* genes, which are known tumor suppressors [ 27, 28], were upregulated. YBX1 has also been reported to be a negative regulator of the p53 pathway [ 29, 30]. Overexpression of the lncRNA *PIK3CD*- *AS2* inhibits the p53 signaling pathway by binding to YBX1, thereby promoting the development of lung cancer and prostate cancer [31]. Our study revealed a novel regulatory axis, *LINC070974*-YBX1-CyclinD1; that is, *LINC070974* knockdown exerts its antitumor effects by retarding YBX1 binding to the *CCND1* promoter ( Figure 8).

Based on these findings, siRNAs targeting *LINC070974* could be designed to treat lung cancer. However, current siRNA delivery methods primarily focus on liver tissue through LNP and GalNAc conjugation; thus, achieving extrahepatic drug delivery is a great challenge [32]. Recently, aerosol inhalation has provided a direct and effective approach for pulmonary drug delivery [33]. In this study, we achieved pulmonary delivery of cholesterol-conjugated siRNA to NSCLC mice via nebulized inhalation and observed a significant inhibitory effect on lung tumor metastasis *in vivo*. In conclusion, our study presents a potential strategy and a feasible technique for effectively treating NSCLC by targeting lncRNAs.

## Supporting information

24152_Supplementary_Table_S4

24152Supplementary_Data

24152Supplementary_Table_S3

## Supplementary Data

Supplementary data is available at *Acta Biochimica et Biophysica Sinica* online.
