# Supplementary material for: Targeting LINC070974 inhibits lung adenocarcinoma cell proliferation and progression by interacting with Y-box binding protein 1 : LINC070974 and YBX1 regulate NSCLC
Source: Acta Biochim Biophys Sin (Shanghai). 2024 Jun 20;57(2):182–94. doi: 10.3724/abbs.2024093 (PMC11868936; doi:10.3724/abbs.2024093)
Supplement: 24152Supplementary_Data [file 24152Supplementary_Data.docx]

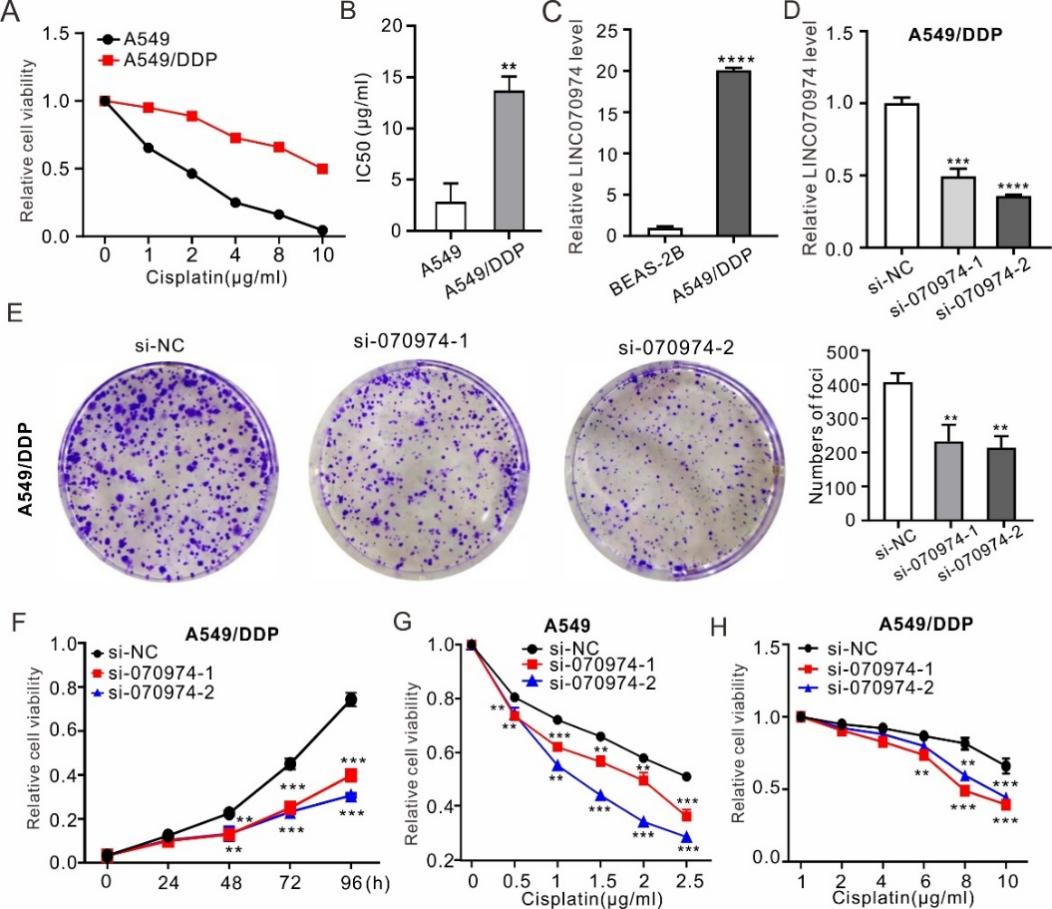


**Supplementary Figure S1. Knockdown of LINC070974 inhibits cell proliferation of cisplatin-resistant A549 cells, related to Figure 1**  (A) Cell viability of A549 cells and A549/DPP cells with cisplatin treatment. (B) Half maximal inhibitory concentrations (IC50) of A549 and A549/DPP cells. (C) LINC070974 expression in BEAS-2B cells (control) and A549/DDP cells detected by qPCR. (D) Validation of siRNA targeting LINC070974 in A549/DDP cells. (E) Colony formation and (F) cell viability assays in A549/DDP cells with LINC070974 knockdown. (G) Effect of cisplatin on cell viability of A549 cells with LINC070974 by CCK-8 assay. (H) Effect of cisplatin on cell viability of A549/DPP cells with LINC070974 knockdown by CCK-8 assay. A549/DPP: cisplatin-resistant non-small lung cancer A549 cell line. ^**^*P* < 0.01, ^***^*P*<0.001, ^****^*P*<0.0001.


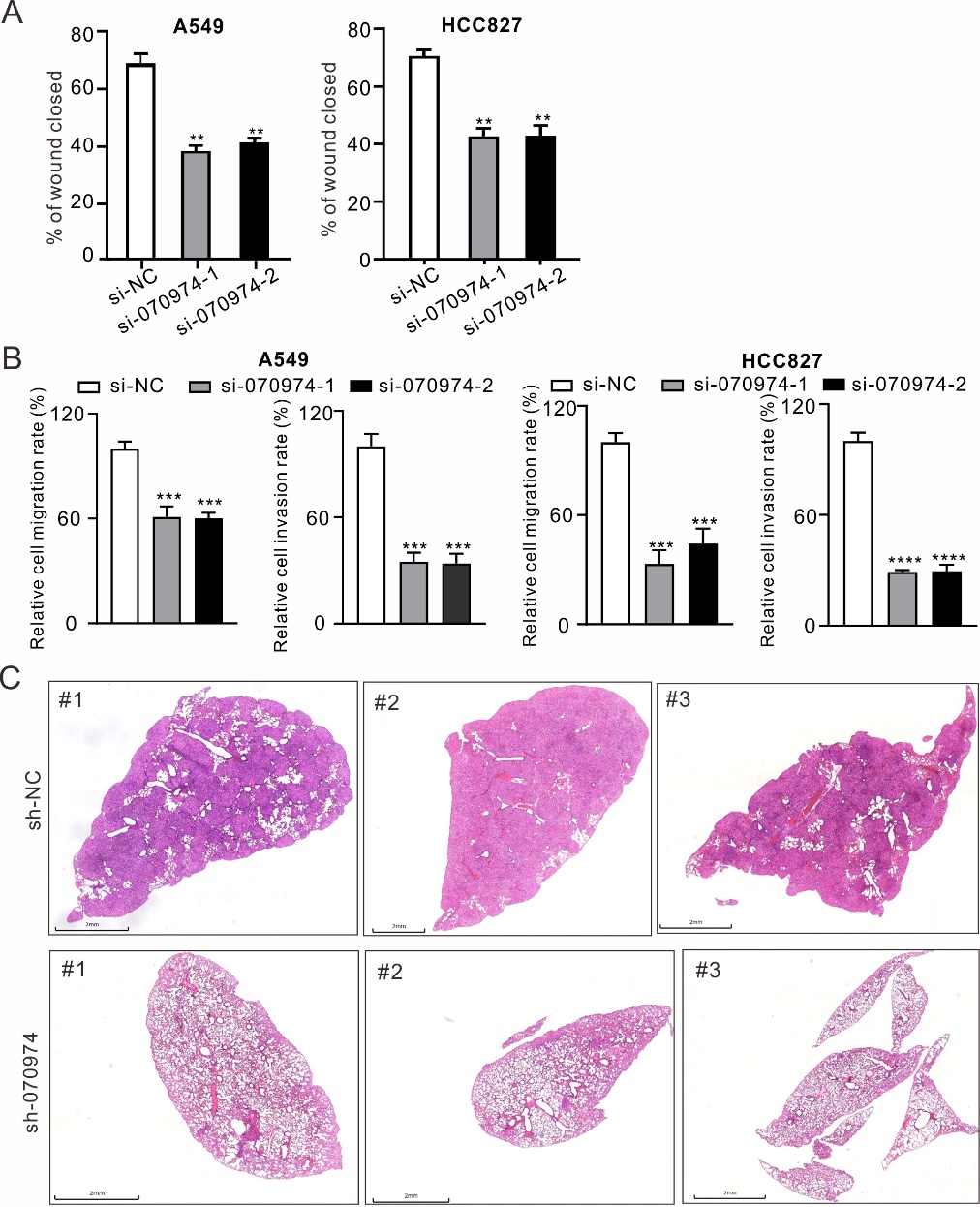


Supplementary Figure S2. **Knockdown of LINC070974 inhibits cell migration and invasion, related to Figure 3**  (A) Wound healing assay of A549 and HCC827 cells with LINC070974 knockdown. (B) Transwell assay. (C) Whole slide imaging of lung tissues with HE staining ^**^*P*<0.01, ^***^*P*<0.001, ^****^*P*<0.0001.


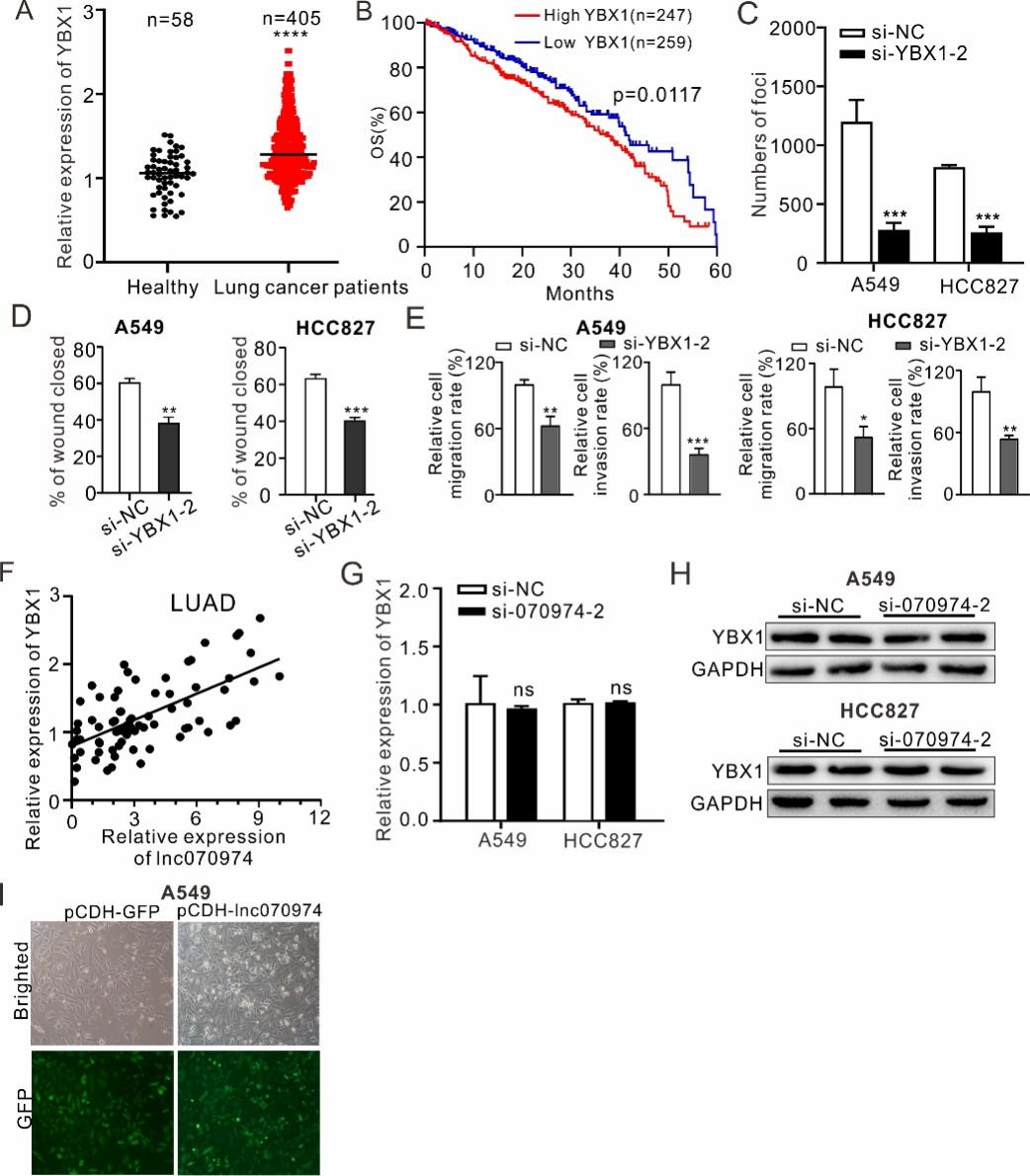


**Supplementary Figure S3. YBX1 is associated with NSCLC and the effect of LINC070974 on YBX1 expression, related to Figure 4**  (A) YBX1 expression in healthy individuals (*n*=58) and lung cancer patients (*n*=405) according to The Cancer Genome Atlas (TCGA) database. (B) Effect of YBX1 expression on overall survival rates. (C) Number of foci on colony formation. (D) Wound healing assay. (E) Transwell assay. (F) Correlation analysis between LINC070974 and YBX1 expression according to TCGA-LUAD dataset. (G) Effect of LINC070974 on YBX1 expression. (H) Effect of LINC070974 on YBX1 protein level. (I) A549 cell line with lentivirus pCDH mediated LINC070974 expression. ^*^*P*<0.05, ^**^*P*<0.01, ^***^*P*<0.001, ^****^*P*<0.0001. ns means no statistically significant.


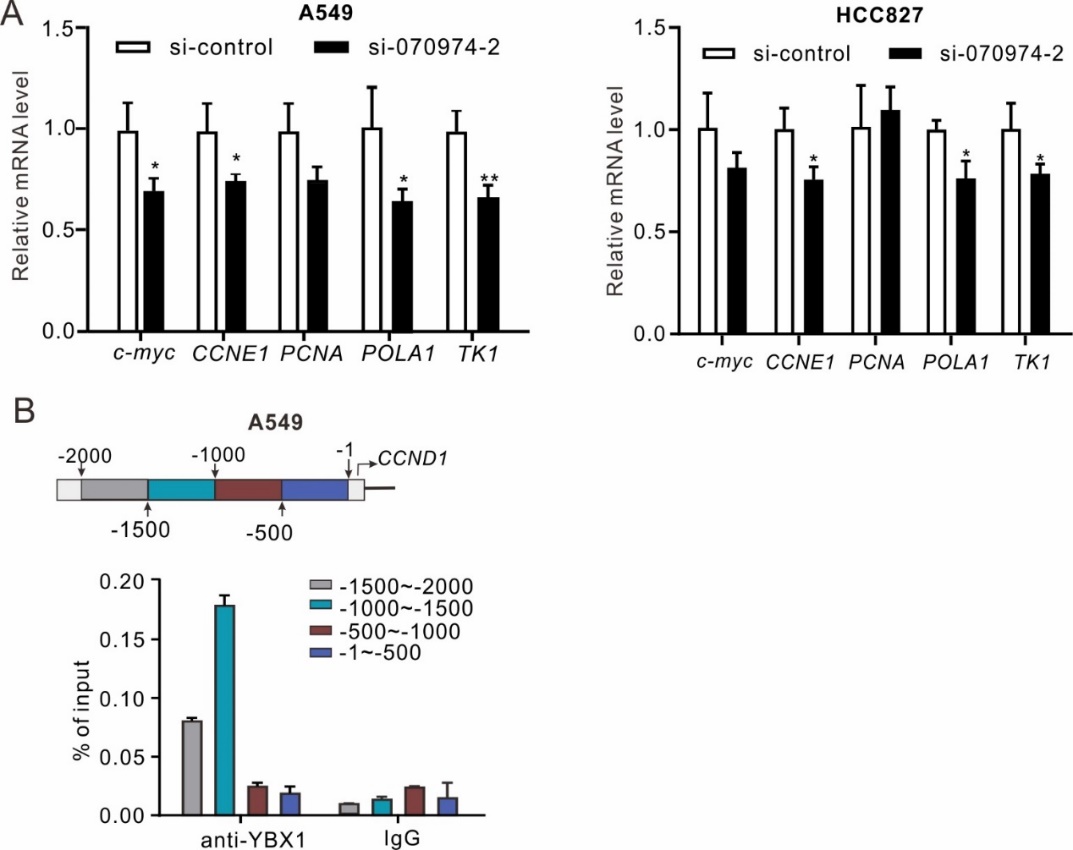


**Supplementary Figure S4. Expression of Rb/E2F1 target genes and binding region of LINC070974 on *CCND1* promoter, related to Figure 6** (A) Expressions of Rb/E2F1 target genes. (B) Binding sites of YBX1 on *CCND1* gene promoter by ChIP-PCR. ^*^*P*<0.05, ^**^*P*<0.01.

| **Supplementary Table S1. Sequences of primers, siRNAs and shRNAs** | |
| --- | --- |
| Primers | Sequences (5′→3′) |
| *LINC070974*-F | GTTGCTTTCCTCGCACGAAC |
| *LINC070974*-R | GTGTGGCTGGTCTGTAGTGG |
| *YBX1*-F | TAGACGCTATCCACGTCGTAG |
| *YBX1*-R | ATCCCTCGTTCTTTTCCCCAC |
| *GAPDH*-F | TGAAGGTCGGAGTCAACGGA |
| *GAPDH*-R | CCTGGAAGATGGTGATGGGAT |
| *β-actin*-F | GGCACCACACCTTCTACAAT |
| *β-actin*-R | GCCTGGATAGCAACGTACAT |
| *CCND1*-F | GTGTA TCGAGAGGCCAAAGG |
| *CCND1*-R | CAACCAGAAA TGCACAGACC |
| *CCNE1*-F | AAGGAGCGGGACACCATGA |
| *CCNE1*-R | ACGGTCACGTTTGCCTTCC |
| *c-myc*-F | AGGGAGATCCGGAGCGAATA |
| *c-myc*-R | GTCCTTGCTCGGGTGTTGT |
| *TK1*-F | GGGCAGATCCAGGTGATTCTC |
| *TK1*-R | TGTAGCGAGTGTCTTTGGCATA |
| *POLA1*-F | ACGCCAGGATGATGACTGGA |
| *POLA1*-R | GTCACTGCGAGCTTCTTTACAT |
| *PCNA*-F | ACACTAAGGGCCGAAGATAACG |
| *PCNA*-R | ACAGCATCTCCAATATGGCTGA |
| *RRM2*-F | GCCACACCATGAATTGTCCG |
| *RRM2*-R | ATGGTAAGTCACAGCCAGCC |
| *DDB2*-F | GTAGTCCCCGCCTTGTTTCT |
| *DDB2*-R | TTTCTGGGCGTTTCTTGGGA |
| *TP54I3*-F | GCTGCTATCCAACTCACCCG |
| *TP53I3*-R | GCATTTGCTTGTACTTTGGTGA |
| *BAX*-F | TGGGCTGGACATTGGACTTC |
| *BAX*-R | GAGACAGGGACATCAGTCGC |
| *PERP*-F | GGACCCCAGATGCTTGTCTT |
| *PERP*-R | TAAGTGACAGCAGGGTTGGC |
| *SESN1*-F | AGCTTCTGGAGGCAGTTCAAG |
| *SESN1*-R | CATTGGTCCTGGGGCTTAGT |
| *BBC3*-F | GGACGACCTCAACGCACAGTA |
| *BBC3*-R | CTAATTGGGCTCCATCTCGGG |
| *CDK6*-F | GCTGACCAGCAGTACGAATG |
| *CDK6*-R | GCACACATCAAACAACCTGACC |
| *CCND1*-2000~1500-F | ATGCGGAATCCGGGGGTAAT |
| *CCND1*-2000~1500-R | CCCGCCGGGAATTAGGATT |
| *CCND1*-1500~1000-F | TGCACCAAAGAGACAGAACCT |
| *CCND1*-1500~1000-R | CGTGGTTACATGAGAGGGTCC |
| *CCND1*-1000~500-F | GGCTGCTGCTGGAATTTTCG |
| *CCND1*-1000~500-R | CAAGTTTCATTCCGGCGCAC |
| *CCND1*-500~0-F | CTCCACCTCACCCCCTAAATC |
| *CCND1*-500~0-R | TGCCCCTGTAGTCCGGTTT |
| siRNA targets | Sequences (5′→3′) |
| Negative Control-sense | UUCUCCGAACGUGUCACGUTT |
| Negative Control-antisense | ACGUGACACGUUCGGAGAATT |
| *LINC070974*-1-sense | CCAUUCUCUAGCAUCUUAATT |
| *LINC070974*-1-antisense | UUAAGAUGCUAGAGAAUGGTT |
| *LINC070974*-2-sense | CCUUCCUGGUUGGCUGUUUTT |
| *LINC070974*-2-antisense | AAACAGCCAACCAGGAAGGTT |
| *YBX1*-sense-1 | GCAGGAGAACAAGGUAGACTT |
| *YBX1*-antisense-1  *YBX1*-sense-2  *YBX1*-antisense-2 | GUCUACCUUGUUCUCCUGCTT  GGAGUUUGAUGUUGUUGAATT  UUCAACAACAUCAAACUCCTT |

| **Supplementary Table S2. The information of antibodies** | | | |
| --- | --- | --- | --- |
| Name | Brand | Catalog No. | Dilution ratio |
| GAPDH | Abcam | ab181602 | 1:1000 (WB) |
| β-Tubulin | Proteintech | 10068 | 1:1000 (WB) |
| Cyclin D1 | Beyotime | AF1183 | 1:1000 (WB) |
| YBX1 | Proteintech | 20339 | 1:1000(WB), 1: 50 (ChIP) |
| Paxillin | Beyotime | AF1477 | 1:1000 (WB) |
| Paxillin(Ser83） | ECM Biosciences | PP1341 | 1:1000 (WB) |
